# Asunaprevir Evokes Hepatocytes Innate Immunity to Restrict the Replication of Hepatitis C and Dengue Virus

**DOI:** 10.3389/fmicb.2017.00668

**Published:** 2017-04-20

**Authors:** Wei-Lun Tsai, Jin-Shiung Cheng, Chih-Wen Shu, Kwok-Hung Lai, Hoi-Hung Chan, Chun-Ching Wu, Jing-Mei Wu, Ping-I Hsu, Raymond T. Chung, Tsung-Hsien Chang

**Affiliations:** ^1^Division of Gastroenterology and Hepatology, Department of Internal Medicine, Kaohsiung Veterans General HospitalKaohsiung, Taiwan; ^2^School of Medicine, National Yang-Ming UniversityTaipei, Taiwan; ^3^Gastrointestinal Unit, Department of Medicine, Massachusetts General Hospital, Harvard UniversityBoston, MA, USA; ^4^Department of Medical Education and Research, Kaohsiung Veterans General HospitalKaohsiung, Taiwan; ^5^Department of Medical Laboratory Science and Biotechnology, Chung Hwa University of Medical TechnologyTainan, Taiwan

**Keywords:** hepatitis C, Dengue virus, innate immunity, interferon type I, asunaprevir

## Abstract

Type I Interferon-mediated innate immunity against *Flaviviridae*, such as Hepatitis C virus (HCV) and Dengue virus (DENV), involves TLR3, RIG-I-like receptor (RLR) and JAK-STAT signal pathways. Asunaprevir is a newly developed HCV protease inhibitor for HCV treatment. Whether, asunaprevir activates innate immunity to restrict viral infection is unclear. Thus, this study investigates the effect of asunaprevir on innate immunity and its influence on HCV and DENV infection. Huh 7.5.1, Hep-G2 cells, JFH-1 infection model, and DENV-2 infection were used for the analysis. The activity of asunaprevir-regulated innate immunity signal pathway was assessed with IFN-β promoter or IFN-stimulated responsive element (ISRE) reporter assays and immunoblotting of key signal proteins. siRNA-mediated MAVS and TRIF knockdown of cells was performed to assess the effect of asunaprevir-regulated innate immunity against HCV and DENV. Asunaprevir treatment activated ISRE and IFN-β promoter-luciferase activities and signaling proteins in the JAK-STAT, MAVS, and TRIF pathways in Huh 7.5.1 cells. Asunaprevir-mediated signaling activation was decreased in MAVS-knockdown cells. Importantly, both RNA and protein levels of DENV-2 NS3 were decreased in asunaprevir-treated Huh 7.5.1 and HepG2 cells. In MAVS-knockdown cells, the restrictive effect of asunaprevir on HCV and DENV was attenuated. Our findings reveal an unexpected activity of asunaprevir, the activation of MAVS dependent innate immunity to restrict HCV and DENV infection.

## Introduction

Currently, as much as 4% of the world's population is chronically infected with hepatitis C virus (HCV; Kao and Chen, 2005). After 20–30 years of infection, 20–30% of chronic HCV patients will develop cirrhosis of liver or hepatocellular carcinoma (Fattovich et al., 2004). An estimated 2.5 billion people are living in areas where dengue is epidemic, leading to 50–100 million human infections each year (WHO, 2009, 2012). Dengue virus (DENV) infection frequently leads to dengue fever, life-threatening dengue hemorrhagic fever (DHF), or dengue shock syndrome (DSS; Gubler, 1989; WHO, 2009, 2012). Despite the tremendous efforts in anti-Dengue viral research, no clinically approved antiviral agents are available and current treatment of DENV infection is only supportive care (Vaughn et al., 2000; Noble et al., 2010).

Resolution of the three-dimensional structures of several HCV proteins, together with the development of replicative cell culture systems, has led to the identification of a number of potential targets for direct-acting antiviral (DAA) agents (Ploss and Dubuisson, 2012; Schaefer and Chung, 2012). It has been confirmed that IFN-free regimens can lead to HCV eradication (Liang and Ghany, 2014). Recent research has shown that dual therapy with asunaprevir, a NS3 inhibitor, and daclatasvir, an NS5A inhibitor, in HCV genotype 1b-infected null responders resulted in an nearly 90% SVR rates (Chayama et al., 2012). In another study, treatment of asunaprevir and dalactasvir resulted in an SVR rate of 90% in genotype 1b treatment naive patients (Manns et al., 2014). IFNα can eradicate HCV through activation of IFN signaling pathway and activation of IFN-stimulated genes (ISGs; Sameul, 2001; Tai and Chung, 2009; Bartenschlager et al., 2012). Whether DAAs exert immune regulatory effects in the treatment of HCV remains unclear.

Innate immunity to HCV and DENV is triggered by activation of cellular sensors that recognize the presence of pathogen-associated molecular patterns (PAMPs). The two key cellular sensors are Toll-like receptor 3 (TLR3), which senses double-stranded RNA (dsRNA) within endosomes, and the RNA helicase retinoic acid-inducible gene I (RIG-I), which senses intracellular double-stranded RNA or single-stranded viral RNA (Wang et al., 2009; Green et al., 2012, 2014; Li et al., 2012; Horner and Gale, 2013). Signaling initiated by TLR3 and RIG-I is transmitted through the adaptor proteins Toll/IL-1 receptor domain-containing adaptor inducing IFN-beta (TRIF) and mitochondrial antiviral-signaling protein (MAVS) respectively, to interact with TNF receptor-associated factor 3 (TRAF3) and converge on the transcription factors, interferon regulatory factor 3 (IRF3) and NF-κB. IRF3 is phosphorylated by IKK-ε and TANK-binding kinase 1 (TBK1), resulting in its dimerization and nuclear translocation.

Activated IRF3 and NF-κB translocate to the nucleus, where they activate IFN-β gene transcription. The secreted type I IFN cytokine stimulates the IFN-α/β receptor (IFNAR-1 and -2) through autocrine or paracrine interaction and then activates the Janus kinase-signal transducer and activator of transcription (JAK-STAT) signaling pathway (Schindler and Plumlee, 2008; Schoggins et al., 2011). The STAT proteins become phosphorylated by the JAK family members JAK-1 and TYK-2. Phosphorylated STAT-1 and STAT-2 recruit IRF9 to form a complex known as IFN-stimulated gene factor 3 (ISGF3), which translocates into the nucleus, binds to IFN-stimulated response elements (ISRE) and induces the transcription of ISGs, many of which are thought to confer antiviral effects including HCV and Dengue virus (Sameul, 2001; Schindler and Plumlee, 2008; Schoggins and Rice, 2011; Schoggins et al., 2011; Fusco et al., 2013).

HCV directs a variety of viral strategies to disrupt host innate immune defenses through its viral proteins (Bartenschlager et al., 2012; Horner and Gale, 2013). The HCV NS3-4A protease specifically cleaves both MAVS and TRIF to inactivate signals initiated by RIG-I and TLR3 for anatomization of innate immunity. We therefore hypothesized that a distinct HCV protease inhibitor can activate innate immunity. This study first investigated the effects of asunaprevir on hepatoma cells, and found interestingly that asunaprevir activates innate immunity in a MAVS dependent manner in hepatoma cells. In view of these findings, the impact of asunaprevir in activation of innate immunity against HCV and Dengue virus infection was examined.

## Materials and methods

### Cells, virus, and reagents

Huh 7.5.1 cells were grown in Dulbecco's Modified Eagle's Medium (DMEM) supplemented with 10% fetal bovine serum (FBS). The infectious JFH1 plasmid was obtained from Dr. Takaji Wakita and inoculated as previously described (Lin et al., 2010). DENV-2 PL046 (Genbank accession # AJ968413.1) isolated from patients with dengue fever was kindly provided by Lin et al. (1998). These viruses were propagated in mosquito cell line C6/36 (ATCC: CRL-1660) grown in RPMI 1640 medium containing 5% FBS. HepG2 cells were purchased from Bioresource Collection and Research Center (BCRC) and grown in DMEM supplemented with 10% FBS. Asunaprevir was obtained from the Bristol-Myers Squibb Company.

### Luciferase assay

To monitor IFN signaling directed by ISRE, plasmids pISRE-luc (500 ng/well) expressing firefly luciferase and pRL-TK (50 ng/well) expressing Renilla luciferase as an internal control were co-transfected in Huh 7.5.1 cells (1 × 10^5^). To monitor IFN-β promoter signaling, pGL-4 IFNβ-Luc (pIFNβ/FLuc) (500 ng/well) expressing firefly luciferase and pRL-TK (50 ng/well) expressing Renilla luciferase as an internal control were used (Chang et al., 2006, 2009). Huh 7.5.1 cells were transfected with Lipofectamine 2000 Transfection Reagent (Invitrogen) following the manufacturer's protocol. Briefly, cells cultured in 12-well plates were transfected with a DNA precipitate containing plasmid DNA (amounts as indicated and had been adjusted by the vector control to be the same among samples of the experimental group). Five hours after transfection, cells were replenished with culture medium, and then incubated for various time points. Relative luciferase activity was assessed by the Promega dual-luciferase reporter assay system (Promega, Madison, WI).

### Immunofluorescence assay

Immunofluorescence staining of HCV core protein in Huh 7.5.1 cells treated with supernatant of JFH-1 infected Huh 7.5.1 cells and Immunofluorescence staining of DENV DV2NS3 protein in Huh 7.5.1 cells treated with supernatant of DENV infected Huh 7.5.1 cells were performed. Huh7.5.1 cells were fixed with 4% paraformaldehyde, permeabilized with 0.5% TritonX-100, and blocked with 3% bovine serum albumin in PBS. The primary antibody was mouse anti-HCV core (Thermoscientific) or anti- DENV DV2NS3 (#YH3304, Yao-Hong Biotechnology, Taipei). The secondary antibody was goat anti–mouse Alexa Fluor 488 (Invitrogen). DAPI was added to the staining to monitor the nuclear structure. Fluorescence signals were observed by fluorescence microscopy (Zeiss, Axcio Observer A1).

### Immunoblotting

Cells were lysed using radioimmune precipitation assay (RIPA) buffer containing 1% NP-40, 0.1% SDS, 10 mM Tris-HCl (pH 7.4), 1 mM EDTA, 150 mM NaCl and protease inhibitor cocktail (Roche), followed by sonification. Proteins were separated by SDS-PAGE with NuPAGE Novex pre-cast 4–12% Bis-Tris gradient gels (Invitrogen, Carlsbad, CA) and transferred to PVDF membranes. The primary antibodies used were anti-STAT1, anti-phospho-STAT1, anti-STAT2, anti-phospho-STAT2, anti-MAVS, anti-TRIF, anti-PKR, anti-IRF3 (Cell Signaling Technology, Inc., Beverly, MA), anti-HCV core, (Thermoscientific), anti-HCV NS3, anti-HCV NS5A and anti-NS5B (Virogen, Watertown, MA), anti-ISG15, anti-MxA and anti-TRIF (Abcam), anti-TRF3 (Santa Cruz), anti-phospho-IRF3 (Origene) and anti-β-actin (Sigma Life Science and Biochemicals, St. Louis, MO). DENV-2 NS3 was detected by a specific monoclonal antibody against NS3 (Yao-Hong Biotechnology). Secondary antibodies were HRP-conjugated ECL donkey anti-rabbit IgG and HRP-conjugated ECL sheep anti-mouse IgG (Amersham Biosciences, Piscataway, NJ). The ECL Western Blotting Detection Kit (Amersham Biosciences, Piscataway, NJ) was employed to detect chemiluminescent signals. Immunoblots shown in each figure are representative of three independent experiments. Densitometry was performed with ImageJ software. Student's *t*-test was used as statistical test.

### Real-time quantitative PCR (qPCR)

Total cellular and viral RNA was isolated post-infection using RNeasy Mini columns (QIAGEN) or Trizol reagent (Invitrogen) and reverse transcribed by random priming with the High Capacity cDNA Reverse Transcription Kit (Applied Biosystems; Foster City, CA). qPCR amplification involved 3 ng cDNA in 10 μl SYBR Green PCR master mix (Applied Biosystems) with 3 μM primers in ABI StepONE Plus Real-Time PCR system (Applied Biosystems). Transcript levels were normalized to that of hypoxanthine phosphoribosyltransferase (HPRT). The primers used in this study are listed in Table 1.

**Table 1:** **List of primer sequences used in quantitative PCR**.

**Target gene**	**Primer**	**Nucleotide sequence**
GAPDH	Forward	5′-CAACTGGTCGTGGACAACCAT-3′
	Reverse	5′-GCACGGACACTCACAATGTTC-3′
JFH1	Forward	5′-TCTGCGGAACCGGTGAGTA-3′
	Reverse	5′-TCAGGCAGTACCACAAGGC-3′
Dengue virus	Forward	5-AGTTGTTAGTCTACGTGGACCGA-3
	Reverse	5′-CGCGTTTCAGCATATTGAAAG-3′

### siRNA and transfection

SMART pool siRNA for MAVS and TRIF for gene knockdown were purchased from Dharmacon. The negative control siRNA was obtained from QIAGEN. Lipofectamine™ RNAiMAX Transfection Reagent (Invitrogen, Carlsbad, CA) was used for siRNA transfection. Protein expression of each knockdowned gene was confirmed by immunoblotting.

## Results

### Asunaprevir activates ISRE reporter activity in Huh 7.5.1 cells

**Figure 1:**
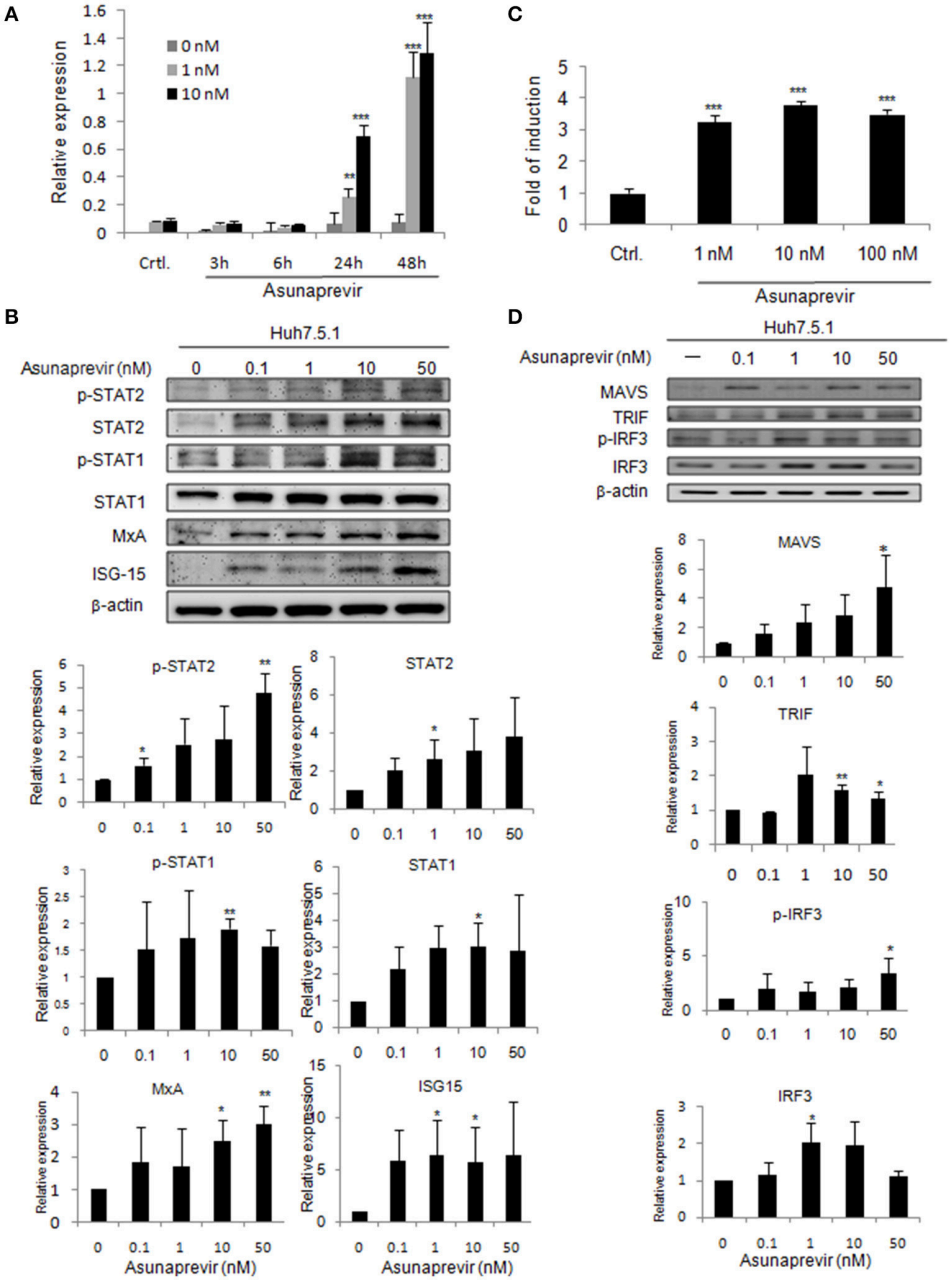
**Asunaprevir activates ISRE activity and type I IFN and TLR3/RIG-I antiviral signaling pathway. (A)** ISRE luciferase reporter assay, plasmids of pISRE-luc expressing firefly luciferase and pRL-TK expressing Renilla luciferase as an internal control were co-transfected to Huh 7.5.1 cells and then treated with 1 or 10 nM of asunaprevir for 3, 6, 24, and 48 h. The 0 nM indicated DMSO vehicle control. The firefly and Renilla luciferase activities were then measured by dual-luciferase assay. Relative firefly luciferase activity was normalized to Renilla luciferase activity. **(B)** Huh 7.5.1 were treated with different doses of asunaprevir for 48 h and the cell lysates were analyzed by immunoblotting with the indicated antibodies involved in the interferon signaling pathway (upper panels). The protein levels of STAT-1, phosphorylated STAT-1, STAT-2, phosphorylated STAT-2, MxA, and ISG-15 relative to the β-actin were determined by densitometry with ImageJ software (lower, panels). **(C)** IFN-β luciferase reporter assay, plasmids pIFN-β/Fluc expressing firefly luciferase and pRL-TK expressing Renilla luciferase as an internal control were co-transfected to Huh 7.5.1 cells and then treated with 1, 10, or 100 nM of asunaprevir for 24 h. Firefly and Renilla luciferase activities were then measured. Relative firefly luciferase activity was normalized to Renilla luciferase activity. **(D)** Huh 7.5.1 cells were treated with different doses of asunaprevir for 48 h and the cell lysates were analyzed by immunoblotting with the indicated antibodies involved in the TLR3/RIG-I signaling pathway (upper panels). The protein levels of MAVS, TRIF, IRF-3, and phosphorylated IRF-3 relative to the β-actin were shown at the bottom of each sample. Immunoblots shown in each figure are representative of three independent experiments. Densitometry was performed with ImageJ software (lower, panels). Values represent the average of three assays ± *S.D*. Statistical significance was tested by Student's *t*-test, ^*^P < 0.05, ^**^P < 0.01, ^***^P < 0.001.

To understand whether asunaprevir activates cellular type-I IFN mediated antiviral machinery, an ISRE-luciferase reporter assay was conducted to assess the STAT1/STAT2 activity of IFN pathway axis. Both pISRE/luc and pRL-TK (transfection control vector) were co-transfected into Huh 7.5.1 cells for 48 h, and then treated with 1 or 10 nM asunaprevir. The luciferase activity was then measured at 3, 6, 24, and 48 h post-treatment. The data showed that Asunaprevir s-stimulated ISRE activity at 24 and 48 h post-treatment in Huh 7.5.1 cells with a dose-dependent manner (Figure 1A).

### Asunaprevir activates type I IFN antiviral signaling pathway

To prove that type I IFN-mediated antiviral signaling is regulated by asunaprevir, the signaling proteins in Huh 7.5.1 cells treated with different doses of Asunaprevir for 48 h were determined by immunoblotting analysis. Results showed that asunaprevir increased STAT1 and STAT2 expression and their phosphorylation in Huh 7.5.1 cells (Figure 1B, upper panels). Downstream ISGs such as MxA and ISG-15 were also induced in asunaprevir-treated cells, demonstrating that asunaprevir activated the JAK-STAT signaling pathway (Figure 1B, upper panels). The protein expression level was also analyzed by densitometry, which showed the consistent results (Figure 1B, lower panels).

### Asunaprevir induces IFN-β reporter activity in Huh 7.5.1 cells

To determine whether asunaprevir induces IFN-β promoter activation with IFN-β promoter luciferase reporter assay, the vectors of pIFN-β/Fluc and pRL-TK were transfected into Huh 7.5.1 cells for 48 h, and then treated with Asunaprevir (1, 10, and 100 nM). After 24 h stimulation, dual luciferase analysis was conducted. Results indicated activation of IFN-β promoter luciferase activity by asunaprevir in Huh 7.5.1 cells (Figure 1C). RNA virus infection-stimulated type I IFN production is dependent on the activation of TLR3 or RIG-I signaling pathway. Because Huh7.5.1 cells are RIG-1 deficient, therefore, this data suggested an unexpected role of asunaprevir in the activation of MAVS- mediated IFN-β production signaling pathway.

### Asunaprevir activates the TLR3 or RIG-I signaling pathway

To understand whether the TLR3 axis is regulated by asunaprevir, the signaling proteins TRIF, IRF-3, and phosphorylated IRF-3 in Huh 7.5.1 cells treated with different doses of asunaprevir for 48 h were determined by immunoblotting analysis, and the protein level was also analyzed by densitometry. Because the activation of MAVS independent of RIG-I was reported (Jacobs and Coyne, 2013), thus, the asunaprevir- induced MAVS was also determined. These results showed that asunaprevir increased MAVS and TRIF expression and also induced the phosphorylation of IRF-3 in Huh 7.5.1 cells (Figure 1D).

### MAVS knockdown abolishes asunaprevir-induced IRF3 activation

**Figure 2:**
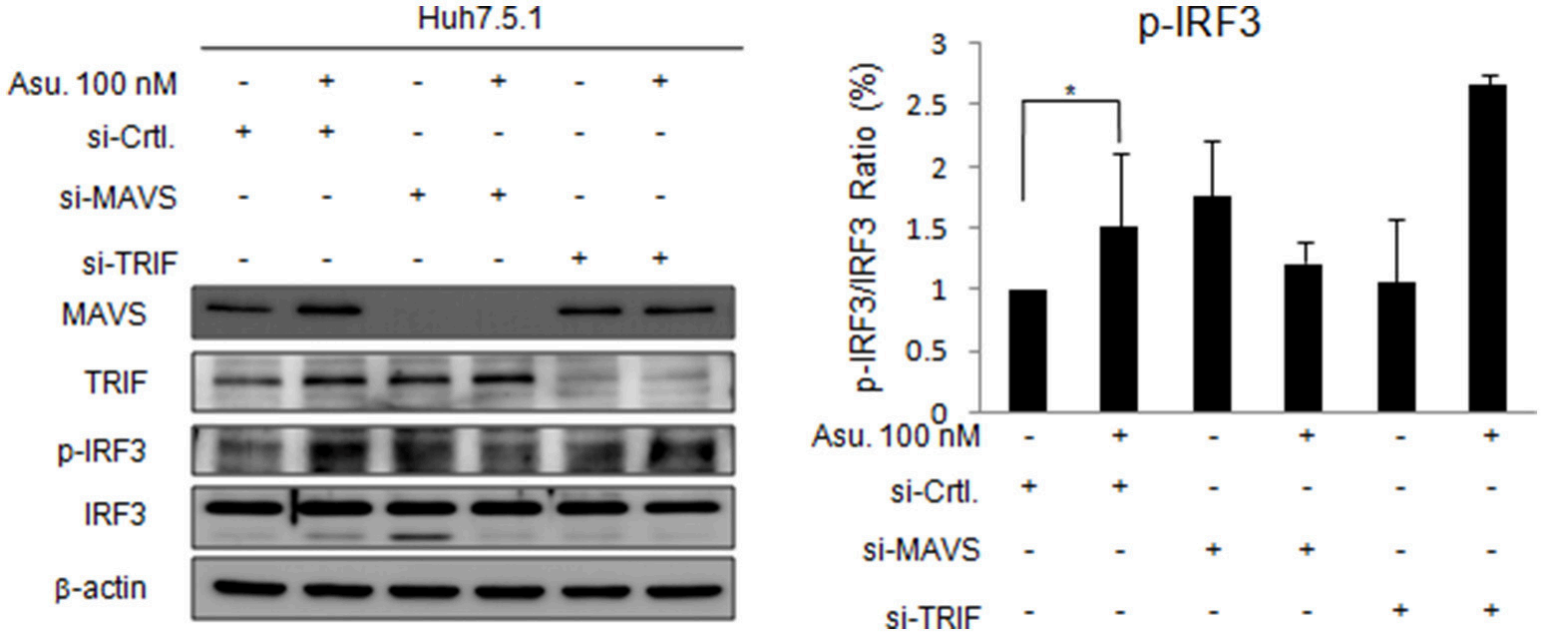
**Effects of Asunaprevir on TLR3/RIG-I signaling pathway in Huh. 7.5.1 cells with MAVS and TRIF knockdown**. Huh 7.5.1 cells were transfected by siRNA of MAVS and TRIF for 48 h and then treated with asunaprevir for 24 h. The key signaling proteins such as MAVS, TRIF, IRF3, and phosphorylated IRF-3 were determined by immunoblotting analysis (left panel). Immunoblots shown in the figure are representative of three independent experiments. The protein levels phosphorylated IRF-3 over total IRF3 was analyzed with ImageJ software (right panel). Data are mean ± *SD* from 3 independent tests. Statistical significance was tested by Student's *t*-test, ^*^P < 0.05.

To validate the role of MAVS and TRIF in asunaprevir-regulated innate immunity, siRNA-mediated knockdown of MAVS and TRIF expression was conducted in Huh 7.5.1 cells. Immunoblotting showed that asunaprevir-induced IRF3 phosphorylation was attenuated in Huh 7.5.1 cells after MAVS knockdown (Figure 2). However, TRIF knockdown was not able to reduce the level of asunaprevir-induced IRF3 phosphorylation. The results indicated that asunaprevir activates innate immune signaling pathway in a MAVS dependent manner.

### Asunaprevir impairs DENV and HCV replication

**Figure 3:**
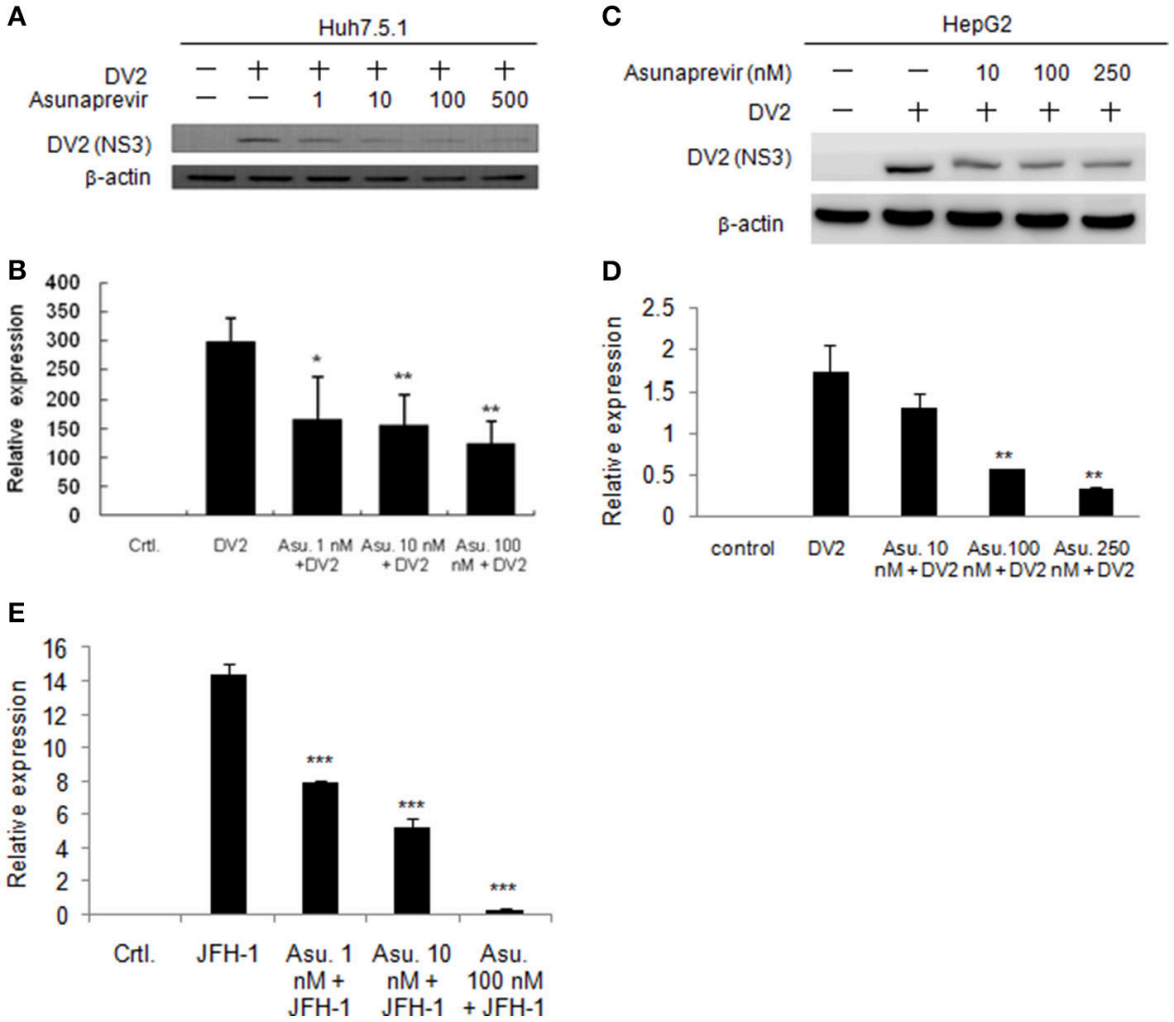
**Effects of Asunaprevir on replication of DENV and HCV. (A)** Huh 7.5.1 cells were treated with different doses of asunaprevir for 24 h and immunoblotting analysis for NS3 protein of DENV, and real-time PCR for RNA level of DENV were performed **(B)**. **(C)** HepG2 cells were treated with different doses of asunaprevir for 24 h and immunoblotting analysis for NS3 protein of DENV, and real-time PCR for RNA level of DENV were performed **(D)**. **(E)** JFH-1 infected Huh 7.5.1 cells were treated with asunaprevir for 24 h, followed by real-time PCR for RNA level of HCV. Data are mean ± *SD* from 3 independent tests. Statistical significance was tested by Student's *t*-test, ^*^P < 0.05, ^**^P < 0.01, ^***^P < 0.001.

As asunaprevir was found to activate host antiviral machinery, the effect of asunaprevir on DENV replication in Huh 7.5.1 and HepG2 cells was assessed. Huh 7.5.1 and HepG2 cells were infected with DENV and treated with asunaprevir for 24 h, followed by immunoblotting analysis for NS3 protein of DENV and real-time PCR for RNA level of DENV-2. After treatment with asunaprevir, the protein level of NS3 and the RNA level of DENV-2 were decreased in Huh 7.5.1 (Figures 3A,B) and HepG2 cells (Figures 3C,D), suggesting that asunaprevir inhibited the replication of DENV in Huh 7.5.1 and HepG2 cells. Asunaprevir is known as a HCV inhibitor. Thus, the anti-HCV effect of Asunaprevir was also determined in our infection model. JFH-1 infected Huh 7.5.1 cells were treated with asunaprevir for 24 h, followed by real-time PCR for RNA level of HCV. The RNA level of HCV was decreased in JFH-1 infected Huh 7.5.1 (Figure 3E).

**Figure 4:**
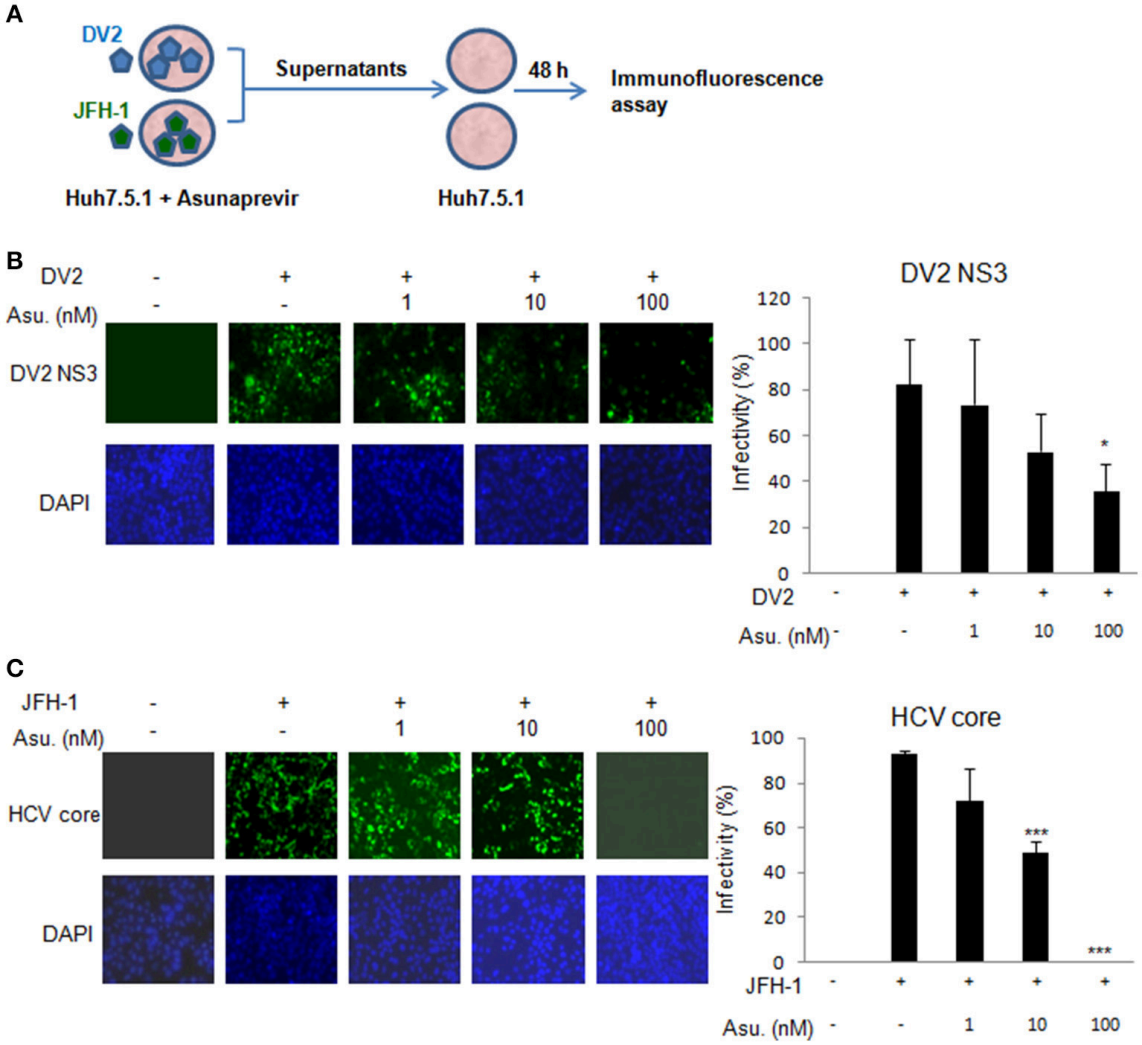
**Asunaprevir inhibits DENV-2 and HCV virus production. (A)** The schematic of asunaprevir treatment in DENV and HCV infection in Huh 7.5.1 cells. The asunaprevir-treated Huh 7.5.1 cells were infected by DENV and JFH-1 for 48 h. The supernatant was harvested and transferred to another Huh 7.5.1 cells. After 48 h incubation, the DENV and HCV-infected cells were determined by immunofluorescence assay. **(B,C)** Immunofluorescence assay, the green fluorescence showed the signals of DENV-2 NS3 protein and HCV core protein, DAPI indicated cell nuclei (left panels). The infectivity of mean ± *SD* was calculated from 3 observation fields (right panels). About 300 cells in each field were inspected. Data are mean ± *SD* from 3 independent tests. Statistical significance was tested by Student's *t*-test, ^*^P < 0.05, ^***^P < 0.001.

Moreover, the Asunaprevir restricted DENV-2 and HCV virus production was demonstrated. The DENV-2 and HCV production was determined using the supernatant from the DENV or JFH-1-infected Huh 7.5.1 cells with Asunaprevir treatment. The supernatant was harvested to infect Huh 7.5.1 cells, after 48 h post infection, the immunofluorescence assay of DENV-NS3 and HCV core proteins was conducted (Figure 4A). The data showed that asunaprevir inhibited the DENV-2 and HCV virus production (Figures 4B,C).

### MAVS activity is involved in the anti-HCV effect of asunaprevir

**Figure 5:**
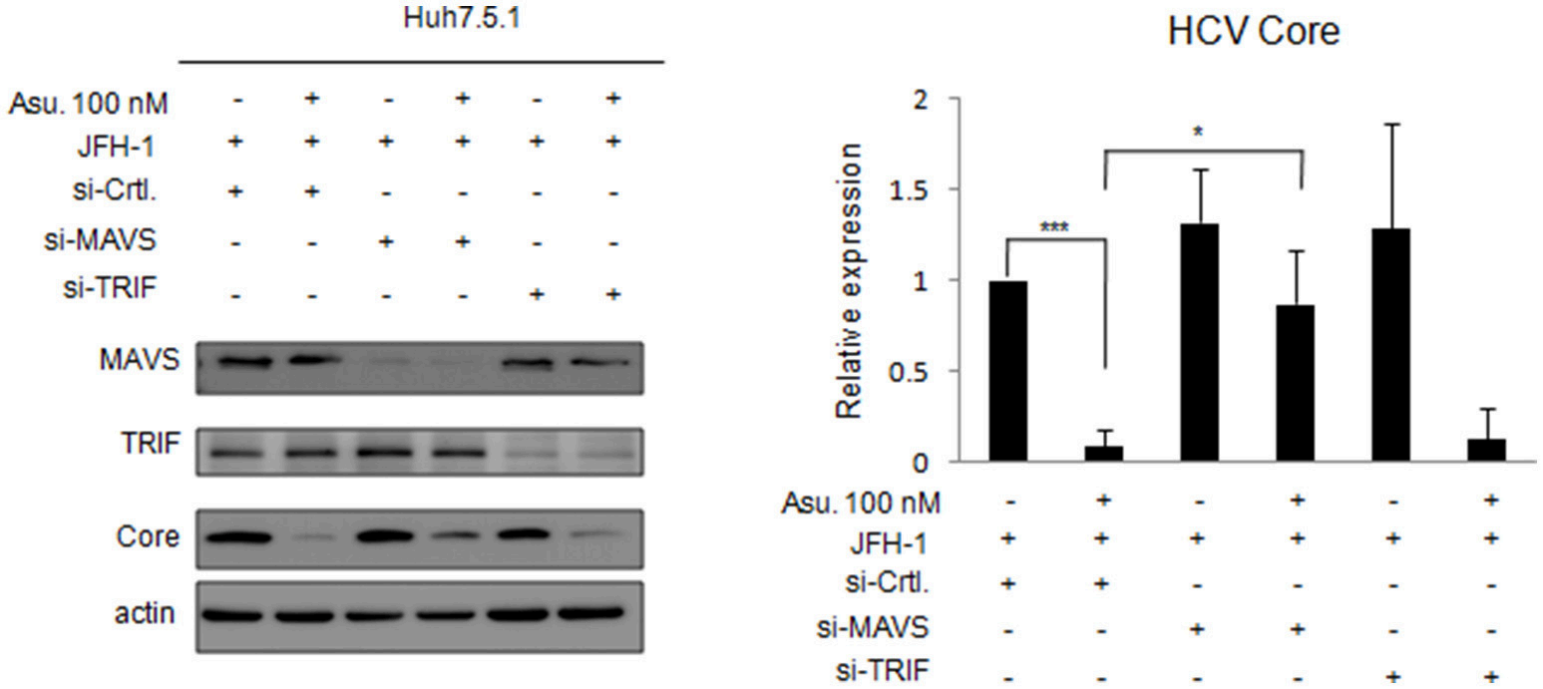
**Effects of asunaprevir on replication of HCV in JFH-1- infected**. Huh 7.5.1 cells after knockdown of MAVS and TRIF by siRNA. JFH-1-infected Huh 7.5.1 cells were transfected by siRNA of MAVS and TRIF for 48 h and then treated with asunaprevir for 24 h. HCV core protein, MAVS and TRIF were determined by immunoblotting analysis. The HCV core protein levels relative to the β-actin were shown at the bottom of each sample. Immunoblots shown in the figure are representative of three independent experiments. Densitometry was performed with ImageJ software. Data are mean ± *SD* from 3 independent tests. Statistical significance was tested by Student's *t*-test, ^*^P < 0.05, ^***^P < 0.001.

Asunaprevir-inhibited HCV replication is rescued in MAVS knockdowned cells. To understand the effect of asunaprevir-activated innate immunity against HCV, both MAVS and TRIF were knocked down in JFH-1-infected Huh 7.5.1 cells, followed by treatment with asunaprevir. HCV core protein, MAVS and TRIF were determined by immunoblotting analysis and the protein expression level was determined by densitometry. After knockdown of MAVS with siRNA, asunaprevir-decreased HCV core protein level was partly rescued in JFH-1-infected Huh 7.5.1 cells (Figure 5). However, the HCV core protein recovery was not shown in TRIF knockdown cells. The data demonstrated that, except directly NS3 inhibition, asunaprevir also inhibit the replication of HCV in JFH-1 infected cells through the activation of MAVS mediated innate immune response additionally.

### Effect of asunaprevir on replication of DENV in Huh 7.5.1 cells with MAVS and TRIF knockdown

**Figure 6:**
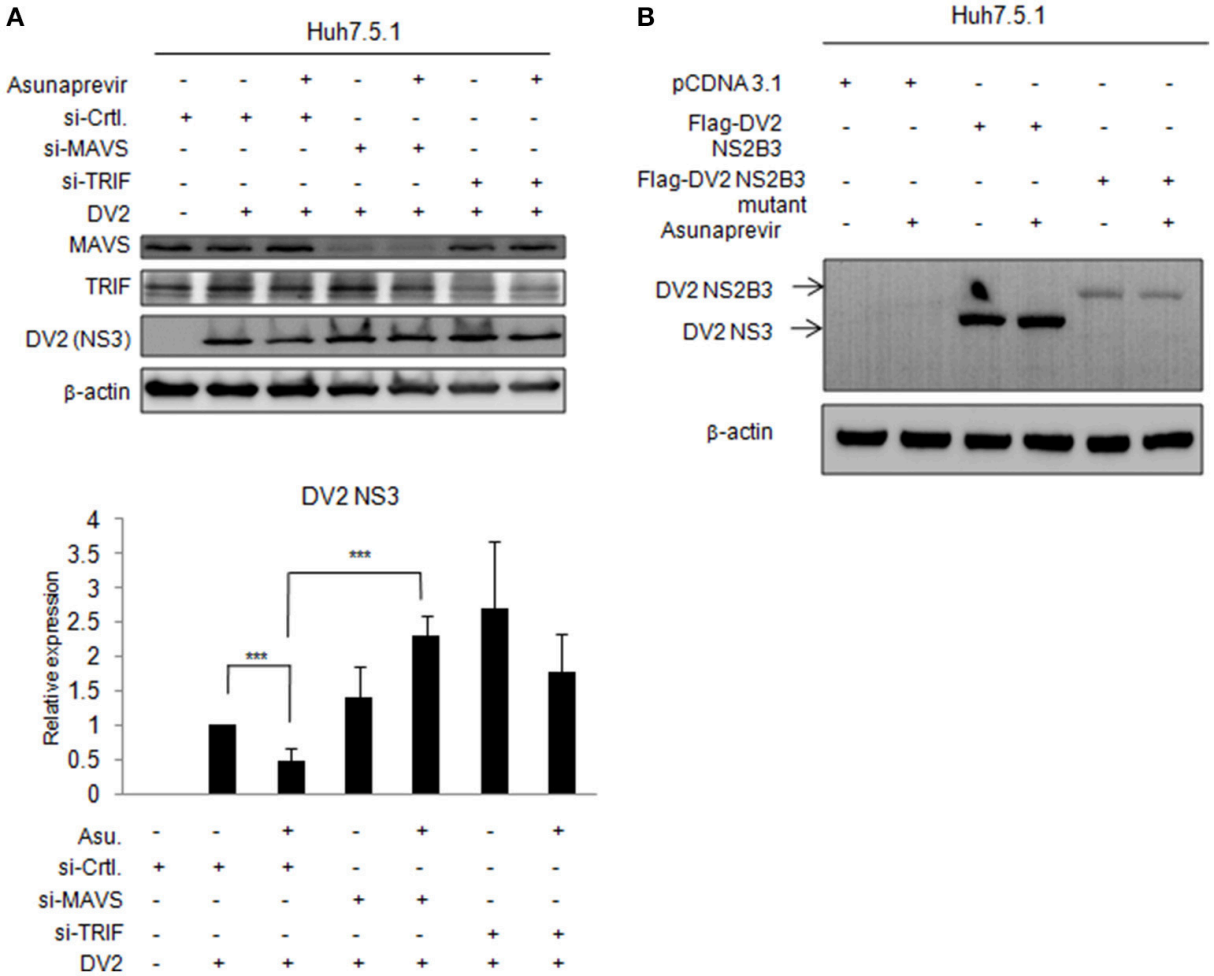
**Effects of asunaprevir on replication of DENV in Huh 7.5.1 cells after knockdown of MAVS and TRIF by siRNA. (A)** Huh 7.5.1 cells infected with DENV were transfected by siRNA of MAVS and TRIF for 48 h and then treated with asunaprevir. Immunoblotting analysis for NS3 protein of DENV, MAVS, and TRIF were determined by immunoblotting Analysis (upper panels). The DENV NS3 protein levels relative to the β-actin were shown at the bottom of each sample. Densitometry was performed with ImageJ software (lower panel). Data are mean ± *SD* from 3 independent tests. Student's *t*-test was used as statistical test, ^***^P < 0.001. **(B)** Huh 7.5.1 cells were transfected with pcDNA3.1 Flag-DV2 NS2B3 or pcDNA3.1 Flag-DV2 NS2B3 protease dead mutant (2 μg) for 24 h and then treated by asunaprevir for 24 h. Immunoblotting was accessed with anti-NS3 and anti β-actin. Immunoblots shown in each figure are representative of three independent experiments.

To understand whether MAVS- and TRIF-mediated innate responses play roles in asunaprevir inhibition against DENV-2 replication, Huh 7.5.1 cells infected with DENV-2 were transfected by siRNA of MAVS and TRIF for 48 h and then treated with asunaprevir. Immunoblotting analysis for NS3 protein of DENV, MAVS, and TRIF showed that asunaprevir-decreased DENV NS3 protein level was rescued in MAVS knockdowned Huh 7.5.1 cells (Figure 6A, upper panels). The densitometry analysis showed consistent results (Figure 6A, lower panel). These data demonstrated that asunaprevir inhibits the replication of DENV in Huh 7.5.1 cells through the activation of the MAVS mediated innate immune response.

### Effect of asunaprevir on NS3 protein of DENV in Huh 7.5.1 cells

Asunaprevir is a HCV protease inhibitor for HCV treatment. To verify whether asunaprevir inhibits DENV protease activity, the Huh7.5.1 cells were transfected with DNEV NS2B3 and treated with asunaprevir. The immunoblots showed the NS3 was processed from NS2B3 by its protease activity, which was not inhibited by asunaprevir (Figure 6B). The NS2B3 protease dead mutant (S135A) was used as the un-cleavage control. Our data confirmed that DENV2 protease was not targeted by asunaprevir. Taking together, our data suggest that asunaprevir elicits host antiviral activity against DENV and HCV beside to HCV protease inhibition.

## Discussion

This study found that asunaprevir activated innate immunity in hepatoma cells. Asunaprevir also restricted the replication of HCV and DENV through the activation of MAVS mediated innate immune response.

It has been recently confirmed that IFN-free regimens can lead to HCV eradication (Liang and Ghany, 2014). Daclatasvir and asunaprevir have received their first global approval in this indication in Japan and is the first all-oral, interferon- and ribavirin-free regimen for this indication (Poole, 2014). In a recent study that investigated the effect of combined dalactasvir and asunaprevir treatment for chronic HCV, Manns et al. (2014) found that baseline dalactasvir, but not asunaprevir, resistance-associated variants are the negative predictors of sustained virological response (SVR; Manns et al., 2014). This finding has the implication that asunaprevir not only possesses a direct antiviral effect but also employs other mechanisms to restrict HCV replication, such that baseline resistance to asunaprevir did not affect the treatment outcome. The present study found that asunaprevir activated innate immunity and inhibited the replication of HCV, which may enhance the direct anti-viral effect of asunaprevir in the treatment of HCV.

HCV directs a variety of strategies to disrupt host innate immune defenses through its viral proteins (Bartenschlager et al., 2012; Horner and Gale, 2013). HCV NS3-4A protease specifically cleaves both MAVS and TRIF to inactivate signals initiated by RIG-I and TLR3 and antagonizes innate immunity (Li K. et al., 2005; Li X. et al., 2005; Meylan et al., 2005; Liu and Gale, 2010). Therefore, it is anticipated that protease inhibitor can activate innate immunity upon inhibition of HCV NS3-4A protease in the context of HCV infection. However, interestingly, this study found that asunaprevir activated the MAVS mediated signaling pathway in Huh 7.5.1 cells not infected by HCV. After knockdown of MAVS but not TRIF with siRNA, the activation of MAVS mediated signaling pathway can be decreased. The present results demonstrated that asunaprevir activated the MAVS mediated signaling pathway in an HCV-independent but MAVS dependent manner. Moreover, after knockdown of MAVS but not TRIF with siRNA, the inhibition of asunaprevir on HCV replication can be partly rescued; evidencing that asunaprevir not only has direct anti-HCV effects but also activates innate immunity and restricts the replication of HCV.

The TLR-3 pathway is closely associated with the type I IFN signal pathway and after knock-down of MAVS, the type I IFN signal pathway also will be blocked (Bartenschlager et al., 2012; Horner and Gale, 2013). We found that asunaprevir treatment activated ISRE and IFN-β promoter-luciferase activities and key signaling proteins in the type I IFN signal pathways in Huh 7.5.1 cells. In MAVS-knockdown cells, the restrictive effects of asunaprevir on HCV and DENV also were attenuated through the blockage of the anti-viral machinery.

Our results found that asunaprevir inhibited the replication of HCV and DENV. The influence of asunaprevir on the replication of HBV was performed, we found that asunaprevir also can inhibit the replication of HBV (Supplementary data Figure S1). We have also explored the effects of several other DAAs on ISRE luciferase activity and found that daclatasvir, MK-5172 and sofosbuvir did not influence the ISRE luciferase activity (Supplementary data Figure S2).

Although Huh 7.5.1 cells were known not to express a functional RIG-I, but activation of MAVS independent of RIG-I is possible (Jacobs and Coyne, 2013). Our results found that asunaprevir increased MAVS expression and MAVS knockdown abolishes asunaprevir-induced MAVS mediated signaling pathway activation. This indicated that asunaprevir may activate the expression of MAVS independent of RIG-I.

Although DENV is an important global health issue, there has been no approved treatment for DENV. DENV can inhibit both type I IFN production and signaling in susceptible human cells. The NS2B3 protease complex of DENV acts an antagonist of type I IFN production. DENV also encodes proteins that antagonize type I IFN signaling, including NS2A, NS4A, NS4B, and NS5 by targeting different components of this signaling pathway, such as STATs. Importantly, the ability of the NS5 protein to bind and degrade STAT2 contributes to the limited host tropism of DENV to humans (Morrison et al., 2012; Green et al., 2014).

Treatment modality that triggers the host defense machinery by innate immune activation can restrict the replication of DENV (Olagnier et al., 2014; Wang et al., 2014) and may be an effective treatment option for DENV infection. Asunaprevir was designed to directly target protease of HCV to inhibit the replication of HCV. Our data confirmed that DENV2 protease was not targeted by asunaprevir. Interestingly, asunaprevir was found to restrict also the replication of DENV in hepatoma cells. Similar to HCV inhibition, the inhibition of asunaprevir on DENV replication can be rescued in MAVS knockdown cells. Hence, asunaprevir restricts the replication of DENV by activating MAVS dependent innate immunity. Further, clinical study is needed to understand the effect of asunaprevir on DENV infection in clinical practice. In conclusion, asunaprevir activates innate immunity and restricts the replication of HCV and DENV through the activation of MAVS mediated innate immune response.

## Author contributions

WT and TC designed the study. TC, WT, JC, HC, KL, CS, PH, and CW carried out data acquisition and analysis. WT and TC wrote the paper. RC and JC supervised the study. All authors reviewed the manuscript.

## Funding

This study was supported by Ministry of Science and Technology of Taiwan, NSC- 102-2314-B-075B-002. Transparency declarations RC reports grants from Gilead, AbbVie, Bristol-Myers Squibb, Merck, Janssen and Mass Biologics. WT reports grants from Gilead.

### Conflict of interest statement

The authors declare that the research was conducted in the absence of any commercial or financial relationships that could be construed as a potential conflict of interest.
