# Supplementary material for: Asunaprevir Evokes Hepatocytes Innate Immunity to Restrict the Replication of Hepatitis C and Dengue Virus
Source: Front Microbiol. 2017 Apr 20;8:668. doi: 10.3389/fmicb.2017.00668 (PMC5397474; doi:10.3389/fmicb.2017.00668)
Supplement: Supplementary file 1 [file DataSheet1.DOC]

**Supplementary data**

**Figure S1**


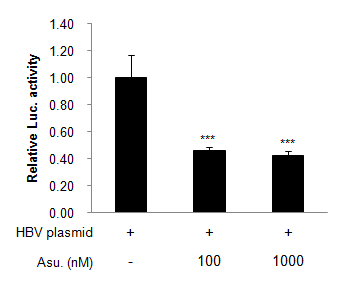


**Figure S1** Huh 7.5.1 cells transfected with plasmid encoding luciferase and HBV fusion gene (pGL3/Fluc-HBV1.2), which is a kind gift from Professor Lin-sheng Zhan, were treated with different doses of asunaprevir and luciferasae activity was analyzed. Data are mean ± SD of three tests. Student’s t test was used as statistical test. *P*-values ***<0.001.

**Figure S2**


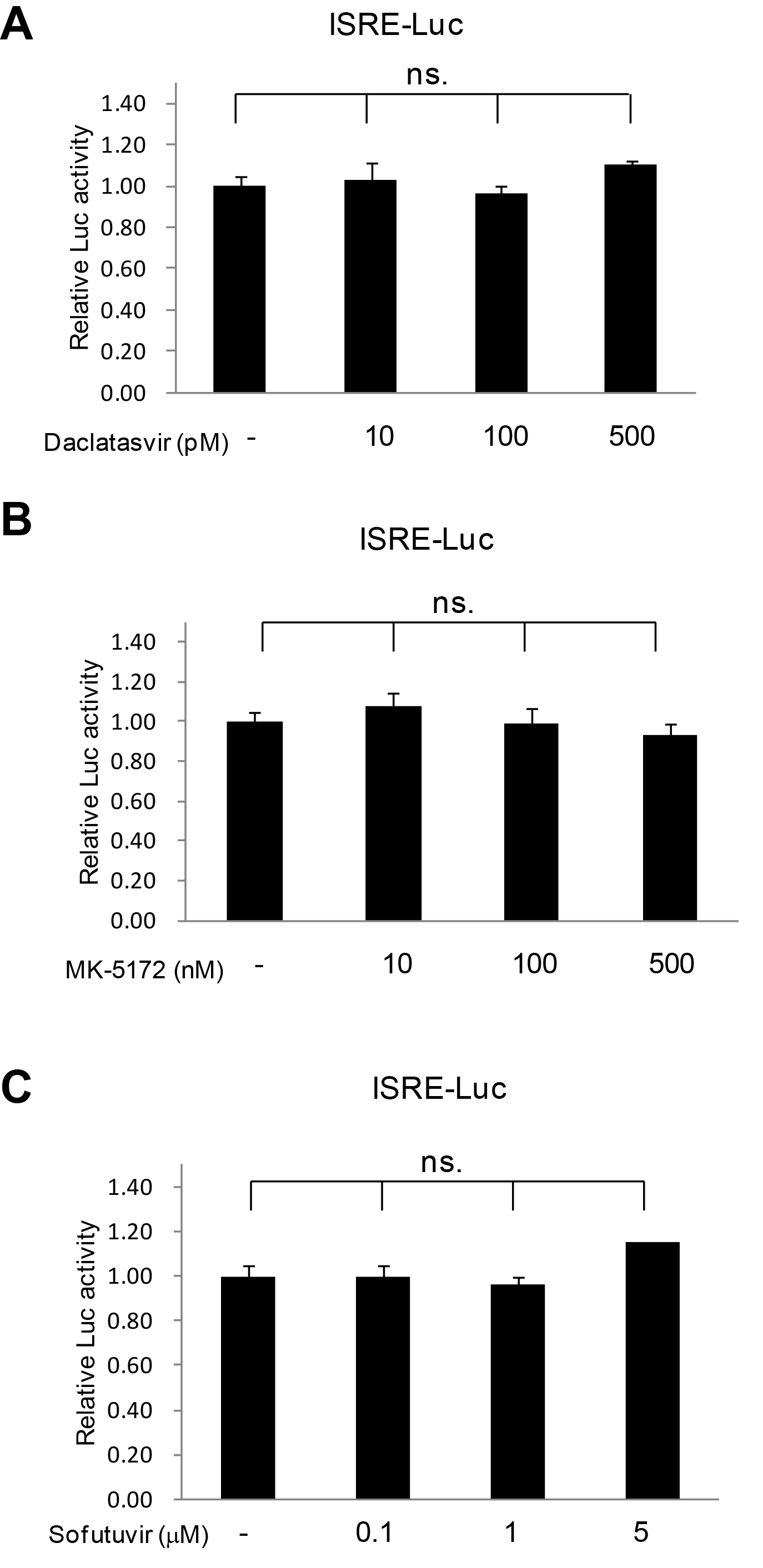


**Figure S2.** Huh 7.5.1 cells were treated with different doses of daclatasvir, MK-5172 and sofosbuvir and the ISRE-luciferase reporter assay was conducted. Data are mean ± SD of three tests. Student’s t test was used as statistical test. The non-significance (ns.) was indicated between control and drugs treatment groups.
